# The role of neutrophils in the upper and lower respiratory tract during influenza virus infection of mice

**DOI:** 10.1186/1465-9921-9-57

**Published:** 2008-08-01

**Authors:** Michelle D Tate, Andrew G Brooks, Patrick C Reading

**Affiliations:** 1 Department of Microbiology and Immunology, The University of Melbourne, Parkville, 3010, Victoria, Australia

## Abstract

**Background:**

Neutrophils have been shown to play a role in host defence against highly virulent and mouse-adapted strains of influenza virus, however it is not clear if an effective neutrophil response is an important factor moderating disease severity during infection with other virus strains. In this study, we have examined the role of neutrophils during infection of mice with influenza virus strain HKx31, a virus strain of the H3N2 subtype and of moderate virulence for mice, to determine the role of neutrophils in the early phase of infection and in clearance of influenza virus from the respiratory tract during the later phase of infection.

**Methods:**

The anti-Gr-1 monoclonal antibody (mAb) RB6-8C5 was used to (i) identify neutrophils in the upper (nasal tissues) and lower (lung) respiratory tract of uninfected and influenza virus-infected mice, and (ii) deplete neutrophils prior to and during influenza virus infection of mice.

**Results:**

Neutrophils were rapidly recruited to the upper and lower airways following influenza virus infection. We demonstrated that use of mAb RB6-8C5 to deplete C57BL/6 (B6) mice of neutrophils is complicated by the ability of this mAb to bind directly to virus-specific CD8^+ ^T cells. Thus, we investigated the role of neutrophils in both the early and later phases of infection using CD8^+ ^T cell-deficient B6.TAP^-/- ^mice. Infection of B6.TAP^-/- ^mice with a low dose of influenza virus did not induce clinical disease in control animals, however RB6-8C5 treatment led to profound weight loss, severe clinical disease and enhanced virus replication throughout the respiratory tract.

**Conclusion:**

Neutrophils play a critical role in limiting influenza virus replication during the early and later phases of infection. Furthermore, a virus strain of moderate virulence can induce severe clinical disease in the absence of an effective neutrophil response.

## Background

Host defence against influenza virus infection depends on a complex interplay of innate (non-specific) and adaptive (specific) components. The development of specific immunity (antibodies and cell-mediated immunity) following infection is of undoubted importance in recovery from infection and resistance to re-infection. Also crucial, however, are pre-existing and rapidly induced innate defence mechanisms which play a critical role in the initial phase of a primary infection, providing a barrier to establishment of a virus and limiting its spread in host tissues in the days before a specific immune response develops. Following inhalation, influenza virus infection is limited to epithelial cells lining the bronchotracheal system, alveolar epithelial cells and airway macrophages [1]. Intranasal administration of mouse-adapted strains of influenza virus leads to development of pneumonia, similar to that observed in human influenza disease. Acute infection is characterized by rapid production of cytokines and chemokines and the subsequent recruitment of inflammatory cells, including macrophages, neutrophils and lymphocytes to the airways. The early inflammatory response aims to limit virus growth in the early phase of infection, prior to the recruitment and activation of virus-specific T lymphocytes (reviewed in [2]).

Although neutrophils have long been considered primary effector cells against bacterial and fungal infections they are also prominent components of inflammatory responses induced by viral infections. Neutrophil infiltration is considered to be a characteristic feature in the early phase of influenza infection in humans, ferrets and mice [3]. Studies using mice irradiated to deplete neutrophils [4] and tumour bearing mice with neutrophilic leukocytosis [5] have suggested that neutrophils can play protective roles against influenza virus infection *in vivo *using virus strain A/PR/8/34. More recently, studies have used anti-granulocyte receptor 1 (Gr-1) mAb RB6-8C5 to examine the contribution of neutrophils to (i) protection in the early phase and (ii) recovery in the late phase following primary infection of mice with highly pathogenic or mouse-adapted strains of influenza A virus [6,7]. Caution must be extended in the interpretation of data obtained following depletion of neutrophils with this mAb, particularly in the latter phase of infection, due to its documented cross-reactivity with additional leukocyte populations, including CD8^+ ^T cells [8-11].

Previous studies have focussed on the role of neutrophils during infection with a virulent recombinant H1N1 virus with genes from the 1918 influenza A virus [7] or with the mouse-adapted PR8 strain [4-6]. Type A influenza viruses of the H3N2 subtype have circulated in the human population since their appearance in 1968 and severe disease is generally associated only with the elderly and/or the immunocompromised. In the mouse model, H3N2 subtype viruses show moderate to low virulence when compared to mouse-adapted virus strains such as PR8 [12]. The current study was undertaken to clarify the role of neutrophils in the upper and lower respiratory tract of mice infected with a H3N2 subtype of moderate virulence for mice. Whilst clear differences are likely to exist between the virulence of a particular virus strain for humans and mice, the mouse serves as a convenient model to gain insight into factors that regulate neutrophil recruitment and the role they play *in vivo*. In initial studies, mAb RB6-8C5 was found to bind to CD8^+ ^T cells and, in particular, to virus-specific CD8^+ ^T cells which could be isolated from the airways at high numbers after day 5 post-infection. Consequently, to investigate the role of neutrophils during the latter stages of infection, CD8^+ ^T cell-deficient B6.TAP^-/- ^mice were treated with RB6-8C5 during infection with a low dose of H3N2 subtype virus. This treatment profoundly enhanced disease and mortality, confirming a critical role for neutrophils in limiting influenza virus replication during the early and later phases of infection.

## Methods

### Mice and viruses

C57BL/6 (B6) mice and TAP-knockout (B6.TAP^-/-^) mice on a B6 background [13] were bred and housed in specific pathogen-free conditions at the Department of Microbiology and Immunology, University of Melbourne, Australia. Adult male mice (6–10 week old) were used in all experiments. The influenza A virus strain used in this study was HKx31 (H3N2), a high-yielding reassortant of A/Aichi/2/68 (H3N2) and A/PR/8/34 (PR8; H1N1) that bears the HA and NA surface glycoproteins of A/Aichi/2/68 and the internal genes of PR8 [14]. Virus was obtained from Alan Hampson, World Health Organization Collaborating Centre for Reference and Research on Influenza, Melbourne, Australia. Virus was grown in 10-day embryonated hen's eggs by standard procedures and titrated on Madin-Darby canine kidney (MDCK) cells as described [15].

### Infection and treatment of mice

Groups of 5 mice were anaesthetized and infected via the intranasal route with 10^5 ^PFU of HKx31 in 50 μl of phosphate buffered saline unless otherwise stated. Each day mice were weighed and scored for clinical illness using the following scale – 0 = no visible signs of disease; 1 = slight ruffling of fur; 2 = ruffled fur, reduced mobility; 3 = ruffled fur, reduced mobility, rapid breathing; 4 = ruffled fur, minimal mobility, huddled appearance, rapid and/or laboured breathing indicative of pneumonia. Animals displaying evidence of pneumonia and/or having lost > 25% of their original body weights were euthanized. All research complied with the University of Melbourne's Animal Experimentation Ethics guidelines and policies. To determine virus titres in organs, mice were euthanized and lungs and nasal tissues were removed, homogenised and clarified by centrifugation. Samples were assayed for infectious virus by standard plaque assay on MDCK cells in the presence of trypsin [15].

To deplete neutrophils *in vivo*, purified anti-Gr-1 rat mAb (RB6-8C5) [16,17] was administered to mice via a combination of intraperitoneal (i.p.; 0.5 mg in 0.2 ml) and intranasal (i.n.; 0.2 mg in 0.05 ml) routes. Mice were treated 1 day prior to infection and every second day thereafter. Control animals received either a similar dose of purified whole rat IgG (Jackson Laboratories, USA) or an equivalent volume of PBS alone. Neutrophil depletion in the blood and the airspaces of the lung was confirmed by differential leukocyte counts as described below.

To deplete CD8^+ ^cells, mice received a single i.p. injection of 1 mg/ml purified mAb YTS169.4 [18] (a kind gift from Assoc. Prof. Andrew Lew and Dr. Yifan Zhan, The Walter and Eliza Hall Institute of Medical Research, Melbourne, Australia) while control mice received an equivalent dose of rat IgG. The effectiveness of CD8 depletion was monitored by flow cytometry analysis of spleen and bronchoalveolar lavage (BAL) cell preparations using PE-labelled mAb 53-6.7, an anti-CD8 mAb that recognizes an epitope distinct from that bound by mAb YTS169.

### Recovery of immune cells

BAL, blood, lung and nasal tissue cells were obtained from uninfected and virus-infected mice. Heparinized blood was obtained by cardiac puncture using a 29-guage needle. For collection of BAL cells, mice were euthanized and the lungs flushed three times with 1 ml of PBS through a blunted 23-guage needle inserted into the trachea. Cells from the nasal tissues (nasal cavity and nasal turbinates) were obtained by cutting down the vertical plane of the skull and scraping out the tissues and small bones from both sides of the nasal passages. Single cell suspensions of lung and nasal tissues were prepared by incubating samples with 2 mg/mL Collagenase A (Roche Diagnostics, Mannheim, Germany) in serum-free RMPI 1640 medium at 37°C for 30 minutes. Each sample was passed through a wire mesh to obtain a single cell suspension. Blood, BAL, lung and nasal tissue samples were treated with Tris-NH_4_Cl (0.14 M NH_4_Cl in 17 mM Tris, adjusted to pH 7.2) to lyse erythrocytes and washed in RPMI 1640 medium supplemented with 10% FCS (RF_10_). Cell viability was assessed via trypan blue exclusion and cell numbers were determined by counting in a hemocytometer.

### Differential leukocyte counts, cell staining and flow cytometry

Leukocytes present in blood, BAL, lung and nasal tissue samples were analyzed by differential leukocyte counts or by flow cytometry. For differential counts, aliquots of approximately 5 × 10^4 ^cells were cytocentrifuged onto glass microscope slides, dried and stained with Diff Quick (Lab Aids, Australia). Slides were examined using a light microscope and a minimum of 100 cells in 4–8 random fields was counted (×1000 magnification). Macrophages, lymphocytes and neutrophils were identified by their distinct nuclear morphologies.

For flow cytometry analysis, single cell suspensions of blood, BAL and nasal tissue cells were incubated on ice for 20 minutes with supernatants from hybridoma 2.4G2 to block Fc receptors and then stained with appropriate combinations of fluorescein isothiocyanate (FITC), phycoerythrin (PE), allophycocyanin (APC) or biotinylated monoclonal antibodies to Gr-1 (RB6-8C5), CD45.2 (104), CD8a (53-6.7), CD4 (GK1.5), B220 (RA3-6B2), NK1.1 (PK136), CD3e (145-2C11), CD11a (M17/4), CD11b (M1/70), CD18 (C71/16), CD49d (MFR4.B), CD62L (MEL-14), CD69 (H1.2F3), CD11c (HL3, all from BD PharMingen), F4/80 (BM8, Caltag Lab.) or CD107a/LAMP-1 (1D4B, a kind gift from Prof. Ken Shortman, Walter and Eliza Hall Institute of Medical Research, Melbourne, Australia). In some experiments, cells were stained with PE or APC-labelled D^b^PA_224 _(SSLENFRAYV)-specific tetramers (a gift from Laureate Prof. Peter Doherty, Department of Microbiology and Immunology, The University of Melbourne) for 60 minutes at room temperature prior to staining with anti-CD8. Propidium iodide (PI; 10 μg/ml) was added to each sample and cells were analysed on a FACS Calibur flow cytometer collecting data from at least 50,000 living (PI^-^) cells. Leukocyte populations were sorted using a MoFlo cell sorter (DakoCytomation, Denmark).

### Measurement of intracellular hydrogen peroxide

Blood and BAL cells were assessed for intracellular hydrogen peroxide (H_2_O_2_) using an established protocol [19]. Fluorescent 6-carboxy-2'7'-dichlorofluorescien diacetate, di(acetoxymethlyl ester) (H_2_DCF; Molecular Probes, USA) is taken up by cells and in the presence of H_2_O_2 _is oxidized to the green florescent product 2'7'-dichlorofluorescein (DCF) which is trapped within the cell and detectable by flow cytometry (FL-1 channel). H_2_DCF (50 μM) was added to cells and incubated for 1 hour at either 4°C or 37°C and DCF levels were determined in Gr-1^high ^cells by flow cytometry.

### Statistical analysis

When comparing two sets of values to each other, Student's *t *test (two-tailed, two-sample equal variance) was used. A *p *value of ≤ 0.05 was considered statistically significant.

## Results

### Rapid and transient neutrophil response in the respiratory tract following influenza virus infection

**Figure 1:**
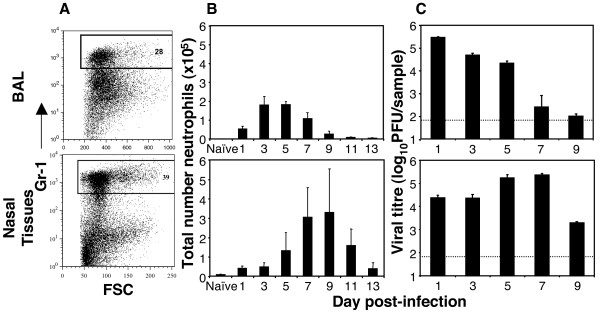
**Neutrophil response in the upper and lower airways following influenza virus infection of mice**. Groups of 5 mice were infected with 10^5 ^PFU of HKx31via the intranasal route. At the times indicated, mice were euthanized and neutrophils in the BAL (upper panels) and nasal tissues (lower panels) were identified as Gr-1^high ^cells by flow cytometry and quantitated. (A) Representative dot plots of Gr-1^high ^cells recovered from BAL and nasal tissues 3 days after infection with HKx31. Graphs show Gr-1 staining of CD45^+ ^leukocytes and the gate used to define Gr-1^high ^neutrophils. (B) The number of neutrophils in BAL and nasal tissues of naïve mice or mice at various times post-infection. A minimum of 50,000 living cells (PI^-^) were collected and analyzed from each mouse. (C) Viral loads in the lung (upper panel) and nasal tissues (lower panel) were determined by plaquing homogenates on MDCK monolayers. Data in panels (B) and (C) show the mean ± 1 SD (n = 5 mice/group). In panel (C), the dotted line represents the detection limit for the plaque assay. The data are representative of 2 or more independent experiments.

A number of studies have identified neutrophils by flow cytometry as Gr-1^high ^inflammatory cells recruited to sites of infection [11,20]. Flow cytometric analysis of cells present in BAL and nasal tissues (Fig. 1A) of mice infected with influenza virus strain HKx31 revealed a population of CD45^+^/Gr-1^high ^cells, a phenotype consistent with that of neutrophils. To confirm the identity of this population, inflammatory cells were recovered from BAL or nasal tissues of virus-infected mice, stained with fluorescent labelled mAbs and the Gr-1^high ^population purified by FACS. Microscopic analysis demonstrated that the Gr-1^high ^population comprised > 90% cells exhibiting a cellular morphology characteristic of neutrophils (data not shown). Gr-1 expression was restricted to inflammatory cells as only cells expressing the common leukocyte antigen CD45 co-stained for Gr-1.

We next examined the kinetics of neutrophil recruitment and accumulation in the respiratory tract following infection with a non-lethal dose of influenza virus strain HKx31. Neutrophils (CD45^+^/Gr-1^high^) were detected at low numbers in the airspaces of uninfected mice where they comprised < 2% of CD45^+ ^BAL cells. Following infection, neutrophil numbers in the BAL increased rapidly, peaking 3–5 days p.i. and declining thereafter (Fig. 1B, upper panel); the neutrophil influx was shown to correlate with a reduction in virus titres in the lung (Fig. 1C, upper panel), consistent with a role in early host defence. Neutrophil accumulation was also observed in the upper airways following influenza virus infection (Fig. 1B, lower panel). Recruitment to the nasal tissues was somewhat delayed compared to the lung, with peak numbers recorded 7–9 days post-infection; again, the timing of the neutrophil influx correlated with the reduction of virus titres in the upper respiratory tract (Fig. 1C, lower panel). Neutrophil recruitment to the airways was a consequence of active viral replication as neither PBS nor UV-inactivated virus induced neutrophil recruitment to the airways of the lung (data not shown). Thus, during a non-lethal influenza virus infection neutrophils were a major component of the inflammatory cell population recruited to the upper and lower respiratory tract.

### Phenotypic analysis of Gr-1^high ^cells from the airways of uninfected and influenza virus-infected mice

**Figure 2:**
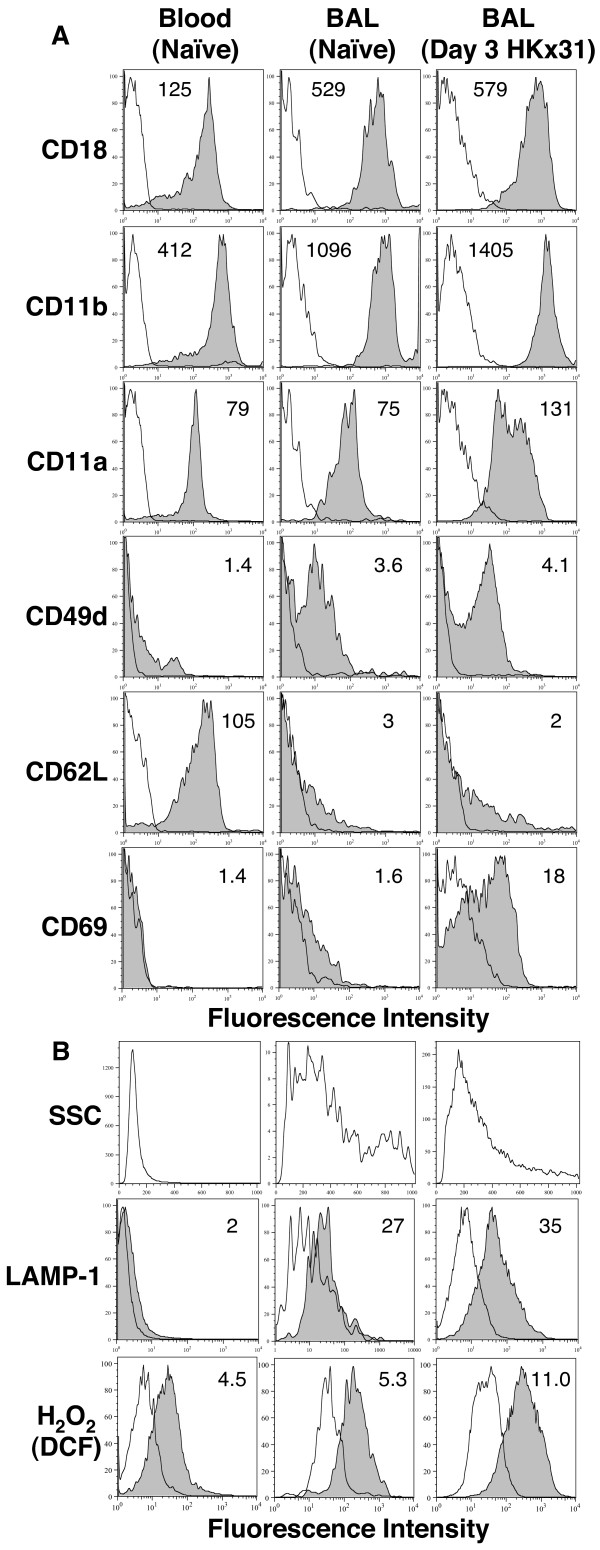
**Expression of adhesion molecules and cell markers by Gr1^high ^neutrophils from naive and influenza virus-infected mice**. Uninfected mice were euthanized and neutrophils in the blood or BAL from pools of 5 mice were examined for expression of cell markers (grey histograms) and appropriate isotype controls (white histograms) via fluorescence intensity. Mice infected with 10^5 ^PFU of HKx31 were euthanized at day 3 post-infection and neutrophils in pooled BAL samples were also examined for expression of (A) cell surface adhesion molecules (B) other markers associated with neutrophil activation including (i) granularity, (ii) cell surface expression of LAMP-1 and (iii) intracellular H_2_O_2 _production. A minimum of 50,000 living cells (PI^-^) were collected and analyzed from each sample and numbers shown beside each histogram represent the MFI of each surface marker on the neutrophil populations indicated, except for H_2_O_2 _production, where numbers shown represent the fold increase in MFI between samples incubated at 4°C and those incubated at 37°C. The data is representative of at least 2 independent experiments.

We next examined a range of phenotypic markers on Gr-1^high ^cells from the blood and BAL of uninfected animals and from the BAL of mice 3 days after infection with HKx31 in an attempt to identify markers associated with neutrophil activation during influenza infection. As neutrophil numbers are particularly low in the BAL from uninfected mice (Fig. 1B), erythrocyte counts confirmed blood contamination to be < 0.01% in all samples analyzed. Adhesion molecules play a critical role in neutrophil recruitment and extravasation from the blood to sites of infection and previous studies have reported modulation of adhesion molecules expressed by pulmonary neutrophils during influenza infection [20]. Our data indicate that CD11b and CD18 are constitutively expressed on blood neutrophils and expression is upregulated on airway neutrophils in the presence or absence of influenza infection (Fig. 2A). CD49d expression is negligible on circulating neutrophils and upregulated on airway neutrophils in a manner that is also independent of viral infection. Conversely, CD62L is expressed at high levels on blood neutrophils but downregulated following migration to the airways of either infected or uninfected mice. Clearly, these data indicate that modulation of many adhesion molecules appears to be associated with recruitment to the airways rather than a consequence of viral infection. A notable exception was CD11a, which in multiple experiments was found to be upregulated on neutrophils from HKx31-infected mice, indicating a degree of virus-specific modulation.

Following a screen of numerous adhesion molecules and cell surface markers (for example, CD31 and CD38, both of which were expressed at low levels on blood and BAL neutrophils) we found CD69 to be the only other marker suitable to discriminate between airway neutrophils from uninfected and virus-infected mice. As seen in Fig. 2A, CD69 is poorly expressed on blood and BAL neutrophils from uninfected mice, but strongly upregulated during viral infection, perhaps reflecting the responsiveness of CD69 to upregulation in the presence of pro-inflammatory cytokines such as IFN-α and IFN-γ [21] which would be produced at high levels in the lungs of infected, but not naïve, mice.

Neutrophils contain antimicrobial products stored in intracellular granules that may be released during infection. Airway neutrophils from infected and uninfected mice were more granular than those recovered from the blood (Fig. 2B), suggesting upregulation of intracellular effector molecules following transmigration to the airways. BAL neutrophils from both infected and uninfected mice also showed enhanced surface expression of lysosomal-associated membrane protein-1 (LAMP-1; Fig. 2B), a protein generally associated with intracellular lysosomal membranes that has been shown to be expressed on the surface of human neutrophils following degranulation [22]. Finally, as reactive oxygen species (ROS) are produced during influenza infections [23], we examined ROS production in murine neutrophils by incubating blood or BAL cells with H_2_DCF which, in the presence of H_2_O_2_, is oxidized to the green fluorescent product DCF [24]. Blood neutrophils and BAL neutrophils from uninfected mice showed a significant increase in the MFI of intracellular DCF generated following incubation at 37°C when compared to control cells incubated at 4°C (Fig. 2B). It is noteworthy that the fold increase in MFI of DCF between 4°C and 37°C was consistently higher in BAL neutrophils from virus-infected mice compared to BAL neutrophils from naïve animals (increases of 1.7, 5.3 and 4.5-fold in BAL neutrophils from uninfected mice compared to 7.6, 11 and 11-fold increases in BAL neutrophils from day 3 HKx31-infected mice in 3 independent experiments), suggesting a higher production of ROS during influenza virus infection.

### Reduced survival and elevated virus titres in neutropenic mice

**Figure 3:**
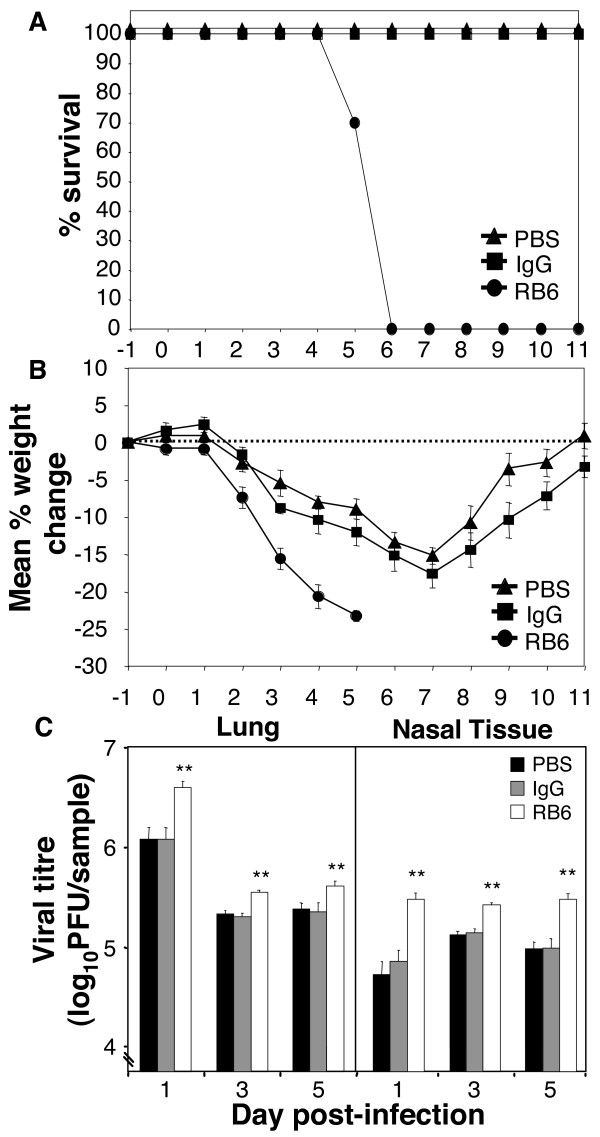
**RB6-8C5 treatment during influenza infection exacerbates disease and leads to increased virus growth in the airways.** Groups of 5 B6 mice were treated with mAb RB6-8C5 (RB6) to deplete neutrophils, or with either rat IgG (IgG) or diluent alone (PBS) 1 day prior to infection with 10^5 ^PFU of HKx31, and every second day thereafter as described in Materials and Methods. (A) Animals displaying evidence of pneumonia and/or having lost > 25% of their original body weights were euthanized. Survival curves were constructed using pooled data from 2 independent experiments (n = 10 mice/group) (B) Mice were weighed daily and results expressed as the mean percent weight change of each group + SEM, compared to the weight immediately prior to infection. (C) Growth and replication of influenza virus in the upper and lower respiratory tract. At various times post-infection, lungs and nasal tissues were removed and levels of infectious virus were assayed in clarified homogenates by plaque assay on MDCK cells. Data represents the mean virus titre ± 1 SD (n = 5 mice/group). ** indicates viral titres of RB6-8C5-treated mice significantly different to those of IgG-treated controls (*p *< 0.01, Student's *t*-test). PBS- and IgG-treated controls were not significantly different to each other at any time-point tested.

To confirm a role for neutrophils in host defence during influenza virus infection we compared survival and virus titres in influenza virus-infected neutropenic (RB6-8C5-treated) and control (PBS- or IgG-treated) mice. We found that a combination of systemic (intraperitoneal) and local (intranasal) treatments was necessary to obtain optimal neutrophil depletion using mAb RB6-8C5 as while systemic depletion alone was sufficient to reduce numbers of blood neutrophils to < 5%, in the absence of an intranasal administration of RB6-8C5, significant numbers of neutrophils accumulated in the airspaces of the lung (data not shown). As seen in Fig. 3A, none of the PBS- or IgG-treated mice infected with HKx31 succumbed to infection over the 11 day monitoring period whereas all virus-infected mice treated with RB6-8C5 were euthanized 5–6 days post-infection. PBS- or IgG-treated mice showed transient weight loss during the first 7 days of infection, after which time mice fully recovered whereas neutropenic mice lost weight rapidly and did not recover (Fig. 3B). Thus, a non-lethal infection becomes lethal following neutrophil depletion of B6 mice.

We next compared virus titres in the upper and lower respiratory tract of infected neutropenic and control mice following infection with HKx31. Virus titres were significantly higher throughout the respiratory tract of RB6-8C5-treated mice at days 1, 3 and 5 when compared to either control group (Fig. 3C). These data suggest that neutrophils contribute to early control of viral replication in the upper and lower respiratory tract and promote survival following infection with a sub-lethal dose of influenza virus.

### RB6-8C5 binds to and depletes CD8^+ ^T cells during influenza virus infection

**Figure 4:**
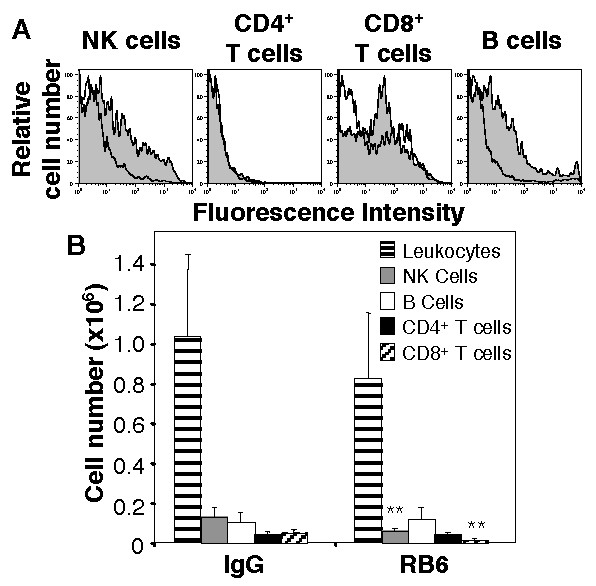
Expression of Gr-1 on lymphocytes recovered from the lungs of influenza virus-infected mice. (A) Binding of fluorescent-labelled RB6-8C5 to NK1.1^+ ^lymphocytes, CD4^+ ^or CD8^+ ^T lymphocytes and B cells (B220^+ ^lymphocytes) from lungs of uninfected mice (white histograms) or mice 3 days after infection with 10^5 ^PFU of HKx31 (grey histograms). Samples represent pooled lung cell suspensions from groups of 3–5 uninfected or virus-infected mice. (B) Cellularity of lung cell suspensions from IgG-treated and RB6-8C5-treated mice 5 days after infection with 10^5 ^PFU of HKx31. Mice treated with either IgG or RB6-8C5 (at days -1, +1 and +3, as described in Materials and Methods) were infected at day 0 with 10^5 ^PFU of HKx31 and lung cell suspensions were prepared from lung tissue at day 5 post-infection. Shown are the mean numbers (± SD) of total leukocytes (CD45^+^), NK cells (NK1.1^+ ^lymphocytes), B cells (B220^+ ^lymphocytes), CD4^+ ^T cells and CD8^+ ^T cells recovered from the lungs of IgG-treated or RB6-8C5-treated mice (n = 5 mice/group). Data are representative of at least 2 independent experiments. ** *p *= 0.015 for NK cells, *p *= 0.017 for CD8^+ ^T cells.

The RB6-8C5 hybridoma was originally described as secreting an antibody specific for granulocyte receptor 1 (Gr-1) [17,25]. Subsequent studies have shown RB6-8C5 to recognize Ly6G, a marker expressed at high levels on granulocytes, and Ly6C expressed by other cell types including subsets of lymphocytes [8,26-28]. To address the possibility that the effects of RB6-8C5 on HKx31-treated mice resulted from cross-reactive depletion of Ly-6C^+ ^cells a number of approaches were taken. First, flow cytometry studies confirmed that Gr-1^high ^cells from the lungs of HKx31-infected mice at day 3 post-infection did not express markers characteristic of B cells (B220^+^), T cells (CD3ε^+^) or NK cells (NK1.1^+^) (data not shown), although intermediate levels of Gr-1 expression were observed on CD8^+ ^T cells and, to a lesser extent, on NK cells and B cells recovered from the lungs of virus-infected mice (Fig. 4A). Second, we examined the effect of RB6-8C5 treatment of mice at day -1, +1 and +3 (relative to infection) on the cellularity of cell suspensions prepared from the lungs of mice 5 days after infection with 10^5 ^PFU of HKx31. Treatment of mice with RB6-8C5 did not reduce numbers of CD4^+ ^T cells or B cells in lung cell suspensions prepared from HKx31-infected mice (Fig. 4B). Of interest, (i) numbers of CD8^+ ^T cells were significantly reduced in the lungs of RB6-8C5-treated mice in multiple experiments (*p *< 0.001 in 2 experiments and *p *< 0.02 in a third), and (ii) numbers of NK cells were significantly reduced in 2 experiments (*p *= 0.015, *p *= 0.022) but not in 3 others (*p *= 0.54, 0.27 and 0.35). The variable effects of RB6-8C5 treatment on NK cells may relate to the intermediate levels of expression observed in this cell population, as seen in Fig 4A.

We have found F4/80 to be a useful marker for identification of resident airway macrophages, however we find this marker to be down-regulated on airway macrophages during influenza infection (data not shown). Therefore, we used cytospin analysis to identify and enumerate monocyte/macrophage populations recovered in the BAL of IgG-treated and RB6-treated mice 5 days after infection with 10^5 ^PFU of HKx31. Whilst a number of studies have reported Gr-1 expression on monocytes and macrophages [29-31], we found that macrophage numbers were not significantly reduced by treatment of mice with anti-Gr-1 antibodies (6.31 × 10^5 ^± 4.61 × 10^4 ^and 8.04 × 10^5 ^± 7.7 × 10^4 ^for IgG-treated and RB6-treated mice, respectively).

**Figure 5:**
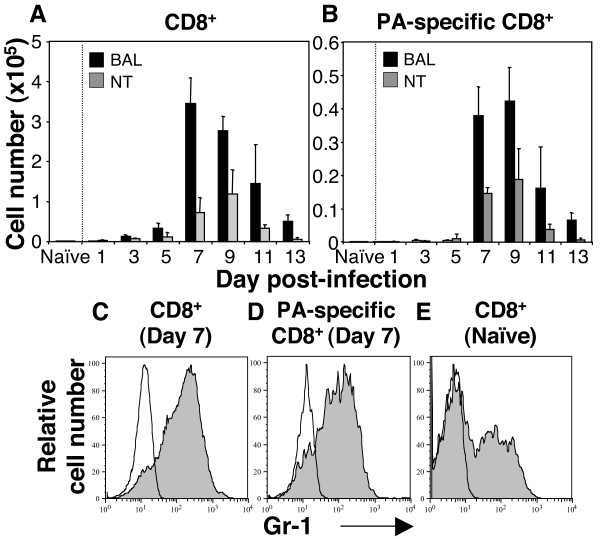
**Recruitment of CD8^+ ^T cells and upregulation of Gr-1 expression during influenza infection.** Groups of 5 mice were infected with 10^5 ^PFU of HKx31 and at various time points post-infection mice were euthanized and BAL cells were analyzed and quantitated by flow cytometry. (A) Total CD8^+ ^T cells and (B) D^b^PA_224_-specific CD8^+ ^T cells are shown. Data are presented as the mean ± SD cell numbers from the BAL or nasal tissues (NT) of uninfected and virus-infected mice. The expression of Gr-1 was determined on (C) CD8^+ ^T cells and (D) D^b^PA_224_-specific CD8^+ ^T cells recovered by BAL at day 7 post-infection, or on (E) CD8^+ ^T cells from the spleen of uninfected mice. Grey histograms represent binding of PE-labelled RB6-8C5 and clear histograms represent binding of an appropriate PE-labelled isotype control. Data shown in (C), (D) and (E) represent a single mouse from a group of 5. All data are representative of 2 or more independent experiments. A minimum of 50,000 living cells (PI^-^) were analyzed for each sample.

It is well documented that virus-specific CD8^+ ^T cells play a critical role in recovery from influenza infection and in resistance to re-infection [2]. Furthermore, depletion of CD8^+ ^T cells *in vivo *following treatment with RB6-8C5 has been reported previously [10,11], although the effects of this mAb on influenza virus-specific CD8^+ ^T cells are unknown. It was therefore of interest to determine the timing of CD8^+ ^T cell recruitment to the airways and the effects of RB6-8C5-treatment on this cell population. First, we determined the kinetics of total (Fig. 5A) and virus-specific (D^b^-PA_224_-tetramer^+^, Fig. 5B) CD8^+ ^T cell recruitment to the upper and lower airways. Numbers of total and D^b^PA_224 _epitope-specific CD8^+ ^T cells were low 1 to 5 days post-infection and increased considerably thereafter, peaking at day 7–9 post-infection. Furthermore, we could demonstrate high levels of direct binding of fluorochrome-labelled RB6-8C5 to both total (Fig. 5C) and D^b^PA_224 _epitope-specific (Fig. 5D) CD8^+ ^T cells isolated from the lungs of HKx31-infected mice when compared to the weaker binding of RB6-8C5 to CD8^+ ^cells recovered from the spleen of uninfected mice (Fig. 5E), indicating that Gr-1 expression is upregulated on airway CD8^+ ^T cells during influenza virus infection.

The effects of RB6-8C5 treatment on CD8^+ ^T cells was unlikely to be a major factor in our studies as all neutropenic mice had succumbed to HKx31 infection by day 5 post-infection (Fig. 3A). To confirm this hypothesis, mice were depleted of CD8^+ ^cells prior to and during infection with HKx31 and the inflammatory response and virus titres determined in the lung. Treatment of mice with anti-CD8^+ ^antibody reduced (i) total CD8^+ ^T cell numbers by > 90% in spleen and BAL, and (ii) D^b^PA_224 _epitope-specific CD8^+ ^T cells by > 95% in BAL at day 5 post-infection but did not reduce neutrophil numbers at either site (data not shown). Moreover, no differences in viral titres were recorded in lung and nasal tissue homogenates of CD8-depleted versus control mice (PBS- or IgG-treated) at days 3 and 5 after infection with HKx31 (data not shown), indicating that the absence of CD8^+ ^cells, including CD8^+ ^T cells, does not have a major impact on early viral replication.

### The effects of RB6-8C5-induced neutropenia during influenza virus infection of mice lacking effective CD8^+ ^T cell immunity

We have confirmed a protective role for neutrophils in the early response to influenza virus infection [6,7], however studies examining their role in viral recovery and clearance are complicated by the cross-reactivity of the RB6-8C5 antibody with virus-specific CD8^+ ^T cells. Therefore, we treated B6.TAP^-/- ^mice, which have defective CD8^+ ^T cell-mediated immunity [13], with RB6-8C5 to induce neutropenia prior to and during infection with HKx31. Preliminary studies established no differences in numbers of BAL neutrophils recovered from HKx31-infected B6 or B6.TAP^-/- ^mice at days 3 and 7 post-infection (data not shown).

**Figure 6:**
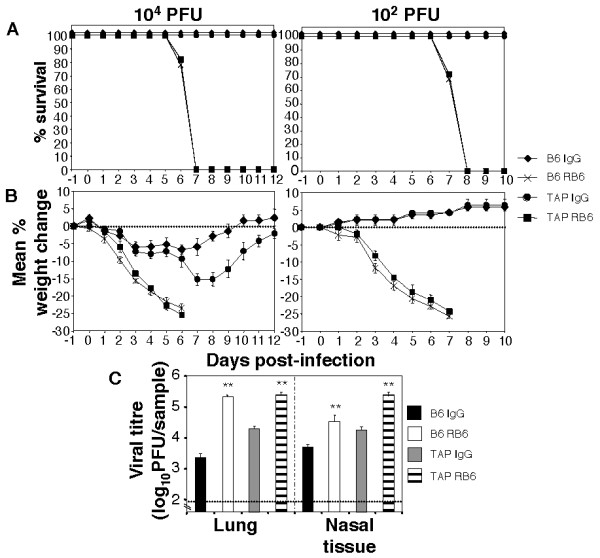
**Effects of RB6-8C5 treatment on influenza infection of CD8^+ ^T cell impaired B6.TAP^-/- ^mice**. Groups of 5 B6 or B6.TAP^-/- ^mice were treated with mAb RB6-8C5 (RB6) to deplete neutrophils, or with either rat IgG (IgG) or diluent alone (PBS) 1 day prior to influenza virus infection and every second day thereafter as described in Materials and Methods. (A) Survival data and (B) weight loss following infection with 10^4 ^PFU (high dose) or 10^2 ^PFU (low dose) of HKx31. Mice were weighed daily and results expressed as the mean percent weight change of each group ± SEM, compared to the weight immediately prior to infection. For survival curves, data has been pooled from 2 independent experiments (n = 10 mice/group) and for weight change data is taken from one of two independent experiments (n = 5 mice/group) (C) Viral growth in the upper and lower respiratory tract 7 days after infection with 10^2 ^PFU of HKx31. Viral loads were determined in homogenates prepared from lungs and nasal tissue at day 7 post-infection by plaque assay on MDCK cell monolayers. Data represent the mean ± 1 SD from 4–5 mice and the dotted line represents the detection limit of the plaque assay. ** indicates viral titres of RB6-8C5-treated mice significantly different to those of the relevant IgG-treated controls (*p *< 0.01, Student's *t*-test).

Initial studies demonstrated that B6.TAP^-/- ^mice were more sensitive to HKx31 infection than immunocompetent B6 animals. Following infection with 10^4 ^PFU of HKx31, survival (Fig. 6A) and weight loss (Fig. 6B) was similar in groups of IgG-treated B6 or B6.TAP^-/- ^mice up to day 6 post-infection, however after this time recovery was significantly delayed in B6.TAP^-/- ^mice. The rapid recovery of B6 mice after day 6 post-infection corresponded with a massive influx of virus-specific CD8^+ ^T cells into the airways at day 7 post-infection (Fig. 5A/B). In contrast, total CD8^+ ^and D^b^PA_224 _epitope-specific CD8^+ ^T cells recovered by BAL from infected B6.TAP^-/- ^mice were < 1% of those from B6 mice at this time (data not shown). Of interest B6.TAP^-/- ^mice did recover from influenza infection, albeit with delayed kinetics, suggesting that other components of adaptive immunity can mediate viral clearance in the absence of effective CTL responses.

The effects of RB6-8C5 treatment on the course of influenza virus infection were compared in B6 and B6.TAP^-/- ^mice using HKx31 at an infectious dose of either 10^4 ^or 10^2 ^PFU. Following infection with 10^4 ^PFU of HKx31, both B6 and B6.TAP^-/- ^mice treated with RB6-8C5 lost weight rapidly compared to their respective IgG-treated controls, with all mice euthanized by day 7 post-infection (Fig. 6A/B). Inoculation of B6 or B6.TAP^-/- ^mice with 10^2 ^PFU of HKx31 led to no significant loss in body weight or signs of disease in IgG-treated controls, yet RB6-8C5 treatment led to rapid and profound weight loss in both B6 and B6.TAP^-/- ^mice and all animals were euthanized 7–8 days post-infection (Fig. 6A/B). Neutrophil-depleted B6 or B6.TAP^-/- ^mice infected with 10^2 ^PFU of HKx31 displayed hunched posture, lethargy and laboured breathing by days 7–8 post-infection (clinical score 3 – 4, as described in Materials and Methods) while IgG-treated controls showed no visible signs of infection (clinical score < 1).

We next examined viral load in the upper and lower respiratory tract of B6 and B6.TAP^-/- ^mice 7 days after infection with 10^2 ^PFU of HKx31. Consistent with the profound impairment of antiviral CD8^+ ^T cell responses observed in B6.TAP^-/- ^mice (see above), virus titres recovered from the lungs and nasal tissues of IgG-treated B6.TAP^-/- ^mice were markedly higher compared to IgG-treated B6 mice (Fig. 6C). Despite these differences, RB6-8C5 treatment of either B6 or B6.TAP^-/- ^mice was associated with significantly higher levels of virus growth throughout the respiratory tract when compared to their respective IgG-treated controls (Fig. 6C). Together, these data indicate that neutrophils play an essential role in the recovery of CD8^+ ^T cell-deficient B6.TAP^-/- ^mice as neutrophil depletion was associated with enhanced virus replication and a profound acceleration of disease in mice infected with a low dose of HKx31.

## Discussion

The results of this study confirm a protective role for neutrophils during influenza infection and establish their critical role in mediating clearance of influenza virus from the respiratory tract of mice infected with a strain of moderate virulence. Treatment with mAb RB6-8C5 was found to induce profound depletion of neutrophils in the blood and throughout the respiratory tract, accompanied by enhanced virus titres and exacerbated morbidity and mortality compared to control mice. A number of observations indicate that our findings are attributable to neutrophil depletion. First, neutrophils were recruited to the airways as early as 8 hrs post-infection (data not shown) and their presence correlated with suppressed virus replication in the airways early after infection. Second, while we could detect direct binding of mAb RB6-8C5 to virus-specific CD8^+ ^T cells, a direct effect on CD8^+ ^T cells is unlikely to account for our results because (i) CD8^+ ^T cell numbers in the airways were particularly low up to day 5 post-infection, (ii) depletion of CD8^+ ^T cells with anti-CD8 mAb YTS169.4 had no effect on viral titres up to and including day 5 post-infection. Furthermore, neutrophil depletion of B6.TAP^-/- ^mice, which have impaired CD8^+ ^T cell immunity, was accompanied by severe disease and enhanced virus replication throughout the airways demonstrating a critical role for neutrophils in mediating viral clearance in this model.

We have demonstrated that neutrophils accumulate in both the upper and lower airways following infection with influenza virus strain HKx31. Initial studies aimed to address the activation status of neutrophils in the airways. Adhesion molecules such as CD11a, CD11b, CD18, CD31, CD38 and CD49d play an important role in neutrophil transmigration [32] and modulation of adhesion molecule expression has been associated with activation during influenza virus infection [20]. We report that much of the modulation observed during influenza virus infection, for example the increased expression of CD11b or CD18 (Fig. 2A), was also observed on the very low numbers of neutrophils recovered from BAL of uninfected mice, suggesting that upregulation is also required for transmigration into the airspaces in the absence of viral infection. Upregulation of CD11a was, however, consistently observed on BAL neutrophils from virus-infected mice compared to naïve controls, suggesting an element of virus-specific modulation. Relative to neutrophils present in the blood, we also found that airway neutrophils from uninfected and infected mice displayed increased granularity, surface expression of LAMP-1 and production of ROS (Fig. 2B), markers typically associated with activation. These findings are consistent with non-specific activation of neutrophils following transmigration into the airways, such that in the absence of infection the low numbers present in the airways are likely activated in response to inhaled stimuli such as dust and other irritants.

Early studies using gamma-irradiated and carrageenan-treated mice suggested a protective role for neutrophils in the early phase of influenza infection [4] and subsequent studies have utilized mAb RB6-8C5 in immunocompetent mice to demonstrate a role for neutrophils in early [6,7] and later-phase protection [6] against highly virulent virus strains. Caution must, however, be exercised interpreting data examining the contribution of neutrophils to protection in the later-phase infection when using immunocompetent mice as we have demonstrated direct binding of mAb RB6-8C5 to virus-specific CD8^+ ^T cells and these cells may also be depleted as a result of RB6-8C5 treatment. A number of factors may contribute to the protective role of neutrophils in the early phase of infection although exact mechanisms underlying their protective effects are yet to be determined. Neutrophils are non-permissive to influenza virus infection [4], adhere to influenza virus-infected cells [33] and mediate phagocytosis of virus-infected, apoptotic cells [34]. In addition, neutrophils have been shown to produce a variety of immune mediators known to display potent antiviral activity against influenza virus, including ROS [23], innate pattern recognition receptors of the defensin [35,36] and pentraxin [37,38] superfamilies and cytokines such as TNF-α [39]. In the late phase of infection, studies have indicated that cooperative interactions between neutrophils and antibody may contribute to protection against influenza infection [6].

Virus-specific CD8^+ ^T cells play an important role in containment and clearance of influenza virus from infected tissue [2]. Thus, any direct effect of mAb RB6-8C5 on CD8^+ ^T cell numbers may contribute to the severe weight loss and disease observed in the late phase of influenza infection in neutropenic mice. In some studies, mAb RB6-8C5 has been reported not to bind CD8^+ ^T cells from naïve animals [40] but others have reported anti-Gr-1 to bind and deplete Ly-6C^+ ^memory-type CD8^+ ^T cells [9]. While anti-Gr-1 antibodies showed minimal cross-reactivity with CD8^+ ^T cells from mice infected with a neurotropic strain of mouse hepatitis virus [11], we clearly demonstrate direct binding of mAb RB6-8C5 to virus-specific CD8^+ ^T cells isolated from the site of influenza virus infection (Fig. 5D). These findings are consistent with direct depletion of CD8^+ ^T cells following RB6-8C5 treatment of influenza virus infected mice and indicate that caution must be exercised when interpreting data obtained using neutrophil depleted mice after day 5 post-infection, following expansion and release of CD8^+ ^T cells in B6 animals.

Despite being somewhat more sensitive to influenza virus, B6.TAP^-/- ^mice were able to clear infection in the absence of effective antiviral CD8^+ ^T cell immunity following inoculation with 10^4 ^PFU of HKx31. Of interest B6.RAG^-/- ^mice, which are entirely deficient of T cells and B cells, were highly sensitive to infection with an equivalent dose of HKx31 such that all mice succumbed to infection 5 days after inoculation (data not shown) arguing that other aspects of adaptive immunity such as CD4^+ ^T cells and/or antibody responses play a critical role in control and clearance of virus from B6.TAP^-/- ^mice. Treatment of influenza virus-infected B6.RAG^-/- ^mice with mAb RB6-8C5 accelerated weight loss and clinical disease such that all mice were euthanized by day 3 post-infection (data not shown) demonstrating that in the absence of adaptive immunity neutrophils play a critical role in restricting early virus replication. The effects of neutrophil depletion could not be studied in later phases of infection due to the exquisite sensitivity of B6.RAG^-/- ^mice to influenza infection.

Treatment of B6 mice or CD8^+ ^T cell-deficient B6.TAP^-/- ^mice with mAb RB6-8C5 induced profound weight loss and clinical disease following infection with 10^4 ^or 10^2 ^PFU of HKx31. It is interesting to note that following infection with a low dose (10^2 ^PFU) of HKx31, weight loss profiles were remarkably similar for RB6-8C5 treated B6 and B6.TAP^-/- ^mice; in either case, virus-infected animals treated with IgG showed no significant weight loss whilst RB6-8C5 treated mice lost weight rapidly and were euthanized by day 7 post-infection. The mechanisms underlying the profound disease phenotype observed in the neutrophil-depleted mice are yet to be fully elucidated. Virus titres recovered from the airways of RB6-8C5-treated mice were 10–100 fold higher than those from control animals at day 7 post-infection (Fig. 6C) and this increased viral burden would certainly be expected to contribute to disease. Although RB6-8C5 treatment has been associated with depletion of a Gr-1^+ ^subset of monocytes [29-31], we used differential leukocyte counts to demonstrate that numbers of inflammatory macrophages/monocytes and lymphocytes recovered from the airways of control and neutrophil-depleted B6.TAP^-/- ^mice were not different at this time were similar (data not shown), arguing that unwanted depletion of additional leukocyte populations was not a major factor contributing to severe clinical disease. In addition, these data also confirm that the morbidity and mortality associated with RB6-8C5 treatment of mice is not due to an overwhelming accumulation of leukocyte populations in the airways.

Inoculation of mice with either a high (10^5 ^PFU) or low (10^2 ^PFU) dose of HKx31 resulted in distinct kinetics of viral replication and this in turn is likely to impact on disease pathogenesis. After inoculation with 10^5 ^PFU of HKx31, virus titres were high early after inoculation and then declined over the course of infection (Fig. 1C). In contrast inoculation with 10^2 ^PFU resulted in increased titres of 10^3^-10^4 ^PFU in the lung at day 7 post-infection (Fig. 6C). Neutrophil depletion of mice infected with either high (Fig. 3) or low (Fig. 6) dose of HKx31 was associated with enhanced viral replication and 100% mortality however viral titres in neutrophil-depleted mice infected with low dose HKx31 at day 7 post-infection (a time associated with a lethal outcome) were similar to those recovered from the lungs of IgG-treated controls infected with a high dose of HKx31 at day 5 post-infection (not associated with a lethal outcome) indicating that viral titres alone do not determine survival during influenza infection. Therefore, while neutrophils play a role in control of viral replication following inoculation with either high or low virus dose, they may also play a critical role in shaping or modulating 'protective' inflammatory responses, perhaps via the secretion of immunomodulatory cytokines and chemokines. Cytokine dysregulation and aberrant innate responses have been proposed to contribute to the pathogenesis of lethal influenza infections [41-43] and could perhaps contribute to the severe disease observed in neutropenic mice.

Although much is known regarding immune responses to influenza in the lung far less is known regarding the effector mechanisms that control viral infection in the nose. Whilst studies have addressed T cell responses in the upper respiratory tract during influenza infection of mice [44-46] the role of innate effectors in the nasal tissues is largely unexplored. Understanding innate responses in the upper respiratory tract is of particular importance as the respiratory epithelium of the nasal mucosa serves as the first susceptible site of influenza infection following inhalation of airborne particles [47]. Clearly, neutrophils are recruited to and accumulate in the upper airways (Fig. 1B) and play a clear role in suppressing viral growth in nasal tissues both early (Fig. 3C) and later (Fig. 6C) in infection. Limiting virus replication in the upper respiratory tract may have important consequences with regard to subsequent transmission of infection to the lung and in limiting viral shedding from the nasopharynx as a result of sneezing. Recent interest regarding transmission of avian H5N1 viruses has highlighted the importance of understanding mechanisms that control virus growth in the upper airways; a single amino acid substitution in the PB2 viral protein was shown to confer avian H5N1 viruses a growth advantage in the upper airways of mice [48] and such a mutation was proposed to potentially provide a platform for adaptation of avian influenza viruses to humans and for efficient person to person transmission. Clearly, understanding immune responses to influenza viruses in the upper respiratory tract has never been more important.

Studies to elucidate the role of neutrophils in murine models of disease are complicated by the absence of a viable 'knockout' mouse that specifically lacks granulocytes. Thus, the anti-Gr-1 mAb RB6-8C5 has been used extensively to characterize the function of Ly6G^+ ^neutrophils in murine models however there is growing evidence to suggest cross-reactivity with the related Ly6C antigen on other leukocytes, including monocytes and lymphocytes [8,26-28]. We have used mAb RB6-8C5 to examine the effects of neutrophil depletion in the early phase of infection using immunocompetent B6 mice, however we have also demonstrated the crucial role of neutrophils in containing the later phases of infection and in mediating viral clearance using B6.TAP^-/- ^mice. Of interest a recent report has described the use of a Ly6G-specific mAb, 1A8, to deplete neutrophils in the absence of direct effects on Gr-1^+ ^monocytes [29]. While the effects of mAb 1A8 on other leukocyte populations such as CD8^+ ^T cells are yet to be determined, memory-type CD8^+ ^T cells have been shown to express Ly6C, but not Ly6G [9]. Similarly, murine plasmacytoid DC are reported to express Ly6C, but not Ly6G [25,49]. Thus, use of a Ly6G-specific mAb to induce neutropenia should allow a more accurate determination of the role of granulocytes in murine models of infection in the absence of unintended effects on other Ly-6C expressing leukocytes.

## Conclusion

Neutrophils were recruited to the upper (nasal tissues) and lower (lung) airways following infection of mice with influenza virus strain HKx31. As mAb RB6-8C5 binds directly to virus-specific CD8^+ ^T cells, we used CD8^+ ^T cell-deficient mice to investigate the role of neutrophils in the early and later phases of influenza virus infection. Neutrophils were shown to play a critical role in limiting virus growth in the upper and lower respiratory tract. Furthermore, neutrophil depletion was associated with profound weight loss and exacerbation of disease in mice infected with a low infectious dose of HKx31. Thus, in the absence of neutrophils, a virus strain that would normally cause mild clinical disease was associated with severe pneumonia, highlighting a critical role for these cells in containment and clearance of influenza infection.

## Competing interests

The authors declare that they have no competing interests.

## Authors' contributions

MT carried out the majority of experiments described in the study, analyzed and interpreted data and participated in the writing of the manuscript. AB contributed to the design of the project, interpretation of data and the writing of the manuscript. PR contributed to all aspects of project design, performed some of the experiments in conjunction with MT and wrote the manuscript. All authors read and approved the final manuscript.
